# Assembly Mechanism of Rhizosphere Fungi in Plant Restoration in Lead Zinc Mining Areas

**DOI:** 10.3390/genes15111398

**Published:** 2024-10-30

**Authors:** Yue Deng, Wenqi Xiao, Zhuang Xiong, Ajia Sha, Yingyong Luo, Xiaodie Chen, Qiang Li

**Affiliations:** 1 School of China Alcoholic Drinks, Luzhou Vocational and Technology College, Luzhou 646000, China; scudeng@hotmail.com; 2 School of Food and Biological Engineering, Chengdu University, Chengdu 610106, China; xwq990713@126.com (W.X.); xiongzhuang2000@126.com (Z.X.); shaajia19980108@126.com (A.S.); lyy1478963@126.com (Y.L.); cxd0512@126.com (X.C.)

**Keywords:** pollution control, bioremediation, microbial diversity, community structure, soil

## Abstract

Background: So far, the assembly and response mechanism of soil fungi in the ecological restoration process of lead zinc mines is still unclear. Methods: In this study, we selected three plants for the ecological restoration of abandoned lead zinc mining areas and explored the community assembly mechanism by which soil fungi assist plants in adapting to the environment during the ecological restoration process. Results: The results revealed that the mining of lead zinc mines led to a significant decrease in soil fungal diversity, whereas the planting of three plants significantly increased the diversity of rhizosphere fungi. Mining activities significantly reduced the abundance of soil *Fusarium*, *Macroventuria*, *Cladosporium*, and *Solicocozyma* and increased the abundance of soil *Helvella*. After three ecologically restored plants were planted, the abundances of *Fusarium* and *Cladosporium* increased significantly, whereas the abundance of *Helvella* decreased significantly. In addition, *Capronia* was significantly enriched in the rhizosphere soils of three plant species in the mining area. β diversity and fungal guild analysis revealed that mining activities had a great impact on fungal communities and guilds. The ecological restoration of plants changed the guilds of rhizosphere fungi, making them closer to those of the control sample. In addition, the endophyte guild was significantly enriched in the rhizosphere soil of three ecologically restored plants, increasing their adaptability. Conclusions: The results provide a reference for screening lead zinc mine bioremediation strains and developing fungal plant joint remediation strategies.

## 1. Introduction

Lead zinc ore, as an important nonferrous metal mineral, plays an important role in industries worldwide. Lead zinc alloys and their products are widely used in various fields, such as automobiles, batteries, construction, and chemicals, and play important roles in promoting global economic development. However, lead and zinc mining has also caused extensive environmental problems [1]. During the mining and smelting process of lead-zinc mines, large amounts of dust and exhaust gas are generated, which are mainly harmful substances such as sulfides and nitrogen oxides. These substances not only have a serious impact on the air quality around mining areas but also pose a potential threat to the global atmospheric environment through atmospheric transmission [2,3]. The mining and smelting process of lead zinc mines also generates wastewater containing heavy metal ions and acidic substances, mainly lead, zinc, and cadmium. If discharged directly into rivers, lakes, and other water bodies without treatment, it poses a threat to aquatic organisms and threatens human health through the food chain [4]. Solid waste, such as waste rocks and residues generated during the mining process of lead zinc mines, can also cause soil pollution [5]. Long-term storage can damage soil structure and fertility, affecting crop growth and yield [6]. Research has shown that lead zinc mining can have a systematic impact on the surrounding ecosystem, leading to a decrease in biodiversity [7,8]. People who are exposed to the surrounding environment of lead and zinc mining for a long period of time face the risk of heavy metal accumulation and irreversible damage to their body tissues [9,10]. An urgent task is to carry out ecological restoration in lead zinc mining areas and reduce their ecological risk.

The ecological restoration and remediation methods for lead zinc mine pollution mainly include physical, chemical, and biological methods [11,12]. Physical methods include precipitation, filtration, adsorption, etc. [13,14]. Chemical methods include neutralization, oxidation reduction, precipitation, etc., all of which can be used to reduce the concentrations of lead and zinc in soil and water [15,16]. However, physical and chemical methods have disadvantages such as high cost and easy secondary pollution. Biological methods rely mainly on the remediation capabilities of plants and microorganisms and have the characteristics of low cost and ecological friendliness [17,18]. Phytoremediation is an in-situ remediation technique that does not cause secondary pollution to ecosystems [19,20]. By planting plants that are adapted to the local environment, vegetation coverage in lead zinc mining areas can be restored, and the soil quality in mining areas can be improved [21,22]. In recent years, plant remediation technology has received widespread attention and research. Researchers have continuously screened and cultivated plants with stronger repair functions to improve plant repair efficiency [23]. Moreover, the combination of plant restoration and other restoration technologies, such as plant microbes combined with restoration technology, has also become a research hotspot [24,25]. Plants and microorganisms achieve efficient removal of pollutants through symbiotic relationships, utilizing the absorption and transformation abilities of plants and the degradation and transformation abilities of microorganisms [26,27]. Microorganisms have the ability to adsorb harmful substances and can remove them from the environment by binding to pollutants through their own surface viscous substances or extracellular polymeric substances [28,29]. Microorganisms can also reduce heavy metal pollutants through metabolic processes, transforming them into harmless forms [30]. In addition, microorganisms can secrete substances that promote plant growth, regulate plant tolerance and adsorption capacity for heavy metals, and improve soil conditions during long-term coevolution and mutual adaptation with plants [31,32]. However, there is currently limited research on the in-situ phytoremediation of lead zinc mines, and the adaptability and remediation capabilities of different plants vary in different environments. The composition and role of different plant rhizosphere fungal communities in the process of phytoremediation are still unknown.

In this study, we planted three types of lead zinc enrichment plants in a lead zinc mining area and adjacent nonmining areas, including *Carex nubigena*, *Pteris cretica* L. var. *nervosa*, and *Neyraudia reynaudiana*. These three plants have been reported to have certain lead zinc enrichment abilities and strong stress resistance and adaptability in local areas [33,34]. After 5 years of cultivation, these ecologically restored plants grew well. To analyze the substrate of the efficient environmental adaptability and remediation ability of these three plants, we analyzed the changes in the composition and diversity of rhizosphere fungal communities in mining and nonmining areas through ITS rRNA high-throughput sequencing. The research results provide data for screening plant growth-promoting bacteria and bioremediation strains and provide application references for ecological restoration in lead zinc mining areas.

## 2. Materials and Methods

### 2.1. Cultivation and Management of Ecologically Restored Plants

The Ya’an lead zinc mine is an abandoned mining area, and it is very important to promote its ecological restoration and reduce the exposure and diffusion risks of pollutants. In 2019, we selected three lead/zinc-enriched plants for the ecological restoration of the Ya’an lead/zinc mining area (Ya’an, Sichuan, China), namely, *C. nubigena*, *P. cretica* L. var. *nervosa*, and *N. reynaudiana*. The three plants are widely present in the local area and have strong tolerance, making them very suitable for the local environment. We randomly planted three types of plants every 1 m in the mining area and adjacent nonmining areas to ensure that the three plants were evenly distributed and that the data were equal in both the mining and nonmining areas. Non-mining areas and mining areas have similar geographical locations, the same altitude, climate, and soil conditions, and no mineral distribution has been found in non-mining areas. Afterward, we watered the plants only 2–3 times within one month after planting to ensure their survival. No additional fertilizers or other exogenous substances were added. Five years later, we collected rhizosphere soils of the three plant species from both mining and nonmining areas for soil fungal diversity sequencing.

### 2.2. Rhizosphere Soil Collection and DNA Extraction

Samples of rhizosphere soil were collected from *C. nubigena*, *P. cretica* L. var. *nervosa*, and *N. reynaudiana* in areas affected by lead zinc mining as well as in nonmining regions. Plants exhibiting consistent growth were first extracted from areas affected by mining activities as well as unaffected areas, after which the soil adhering to their roots was carefully collected by shaking them into self-sealing bags [35]. Each plant provided approximately 200 g of soil for the sequencing of fungal diversity. Moreover, bulk soil samples were procured from both mining and nonmining sites for comparative purposes, with each sample consisting of three biological replicates. The rhizosphere soils of *C. nubigena*, *P. cretica* L. var. *nervosa*, and *N. reynaudiana* collected from the mining area, together with the control soil within the mining area, were denoted as Mine. C, Mine. P, Mine. N, and Mine, respectively. Similarly, the rhizosphere soils of these plant species from nonmining areas, along with their respective control soil samples, were identified as the control. C, control. P, control. N, and control. Each soil sample was replicated three times (*n* = 3), with 50 g of each sample used for fungal diversity analysis and isolation. The 24 samples were transported to the laboratory in an ice bag for DNA extraction and ITS rRNA sequencing. Genomic DNA was extracted from the soil samples via an Omega soil DNA kit (Omega Biotek, Norcross, GA, USA), and the quality of the extracted DNA was evaluated via 1% agarose gels [36].

### 2.3. PCR Amplification and Amplicon Detection

The genomic DNA was diluted to 1 ng/µL with sterile water, followed by amplification of the ITS1 rRNA regions from the samples with primers containing a barcode (ITS5-1737F: 5′-GGAAGTAAAAGTCGTAACAAGG-3′; ITS2-2043R: 5′-GCTGCGTTCTTCATCGATGC-3′). PCR was conducted via the use of 15 µL of Phusion^®^ High-Fidelity PCR Master Mix from New England Biolabs (NEB, Ipswich, MA, USA), 2 µM forward and reverse primers, and 10 ng of template DNA. The thermal cycling protocol commenced with an initial denaturation step at 98 °C for 1 min, followed by 30 cycles consisting of denaturation at 98 °C for 10 s, annealing at 50 °C for 30 s, elongation at 72 °C for 30 s, and a final extension at 72 °C for 5 min. The PCR products were mixed with an equal volume of 1× loading buffer, which included SYBR Green, and separated on a 2% (*w*/*v*) agarose gel for detection. The PCR products were subsequently purified via the Qiagen Gel Extraction Kit manufactured by Qiagen in Germany [37].

### 2.4. Library Preparation, Sequencing, and Data Processing

Sequencing libraries were generated with index codes following the manufacturer’s instructions for the TruSeq^®^ DNA PCR-Free Sample Preparation Kit (Illumina, San Diego, CA, USA). The libraries were evaluated via a Qubit@ 2.0 fluorometer (Thermo Scientific, Waltham, MA, USA) and an Agilent Bioanalyzer 2100 system. Following this assessment, sequencing of the libraries was performed on the Illumina NovaSeq platform, which produced 250-bp paired-end reads. The barcodes and primer sequences were excised from the reads, and subsequently, the reads were merged via FLASH V1.2.7 [38]. To ensure a high-quality outcome, the raw tags were processed through the quality control protocol of QIIME V2 [39]. Through comparison with the reference Silva database, chimera sequences were identified and subsequently excluded from the datasets [40].

### 2.5. OTU Cluster and Species Annotation

Operational taxonomic units (OTUs) were identified by selecting sequences that shared a minimum similarity of 97% via Uparse v7.0.1001 [41]. To provide a more comprehensive characterization of individual OTUs, a representative sequence was identified and annotated through the utilization of the Silva database and the Mothur algorithm [40]. To examine the phylogenetic relationships among the operational taxonomic units (OTUs) and the diversity of dominant species within different samples or groups, multiple sequence alignments were carried out via MUSCLE v3.8.3 [42]. To standardize the abundance data of operational taxonomic units (OTUs), a reference sequence count was established on the basis of the sample containing the fewest sequences. The standardized data were subsequently utilized for assessing both α and β diversity [37].

### 2.6. α and β Diversity Analyses

To assess the complexity of species diversity within a specific sample, six indices were employed, comprising the observed species, Chao1, Shannon, Simpson, ACE, and Good’s coverage indices. The calculation of these indices was performed via QIIME v2 [39], followed by visualization via R v2.15.3. Three indices were selected to evaluate community richness: observed species and the Chao1 estimator. Community diversity was assessed via the Shannon and Simpson indices. β diversity analysis was conducted to compare species complexity among samples. Principal component analysis (PCA) and nonmetric multidimensional scaling (NMDS) were performed via the R vegan software package v4.4.1 [36].

### 2.7. Guild Prediction and Fungal Correlation Analysis

The guilds and trophic modes of the soil fungi were predicted via the FUNGuild algorithm [43]. To reduce the number of variables, we employed the FactoMineR and ggplot2 packages within R software version 2.15.3 to conduct principal component analysis (PCA) [44].

### 2.8. Statistical Analysis

Statistical analysis was carried out on the samples to determine the significance of differences among them using R v2.15.3. The *t* test was used to compare two sets of samples, while Tukey’s test was employed for more than two sets. A P value lower than 0.05 was considered to indicate a statistically significant difference between various groups.

## 3. Results

### 3.1. Sequencing Data Analysis

**Figure 1:**
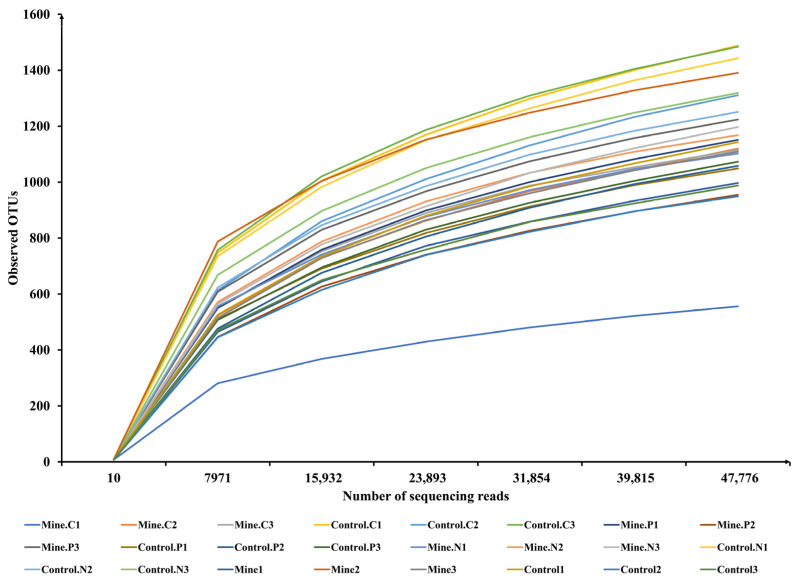
Rarefaction curve of ITS high-throughput sequences.

In this study, we analyzed the assembly mechanism of different plant rhizosphere fungal communities, including *C. nubigena*, *P. cretica* L. var. *nervosa*, and *N. reynaudiana*, during the ecological restoration process in lead zinc mining areas. An average of 86,187 raw reads were obtained per sample (Supplementary Table S1). After removing chimeras, low-quality sequences, and short sequences, we obtained an average of 79,368 effective reads per sample for downstream analysis. We counted the changes in the observed species with the sequencing reads via rarefaction curves (Figure 1). The number of observed OTUs gradually increased with the increasing number of sequencing reads. When the number of sequencing reads exceeded 31,854, the rarefaction curves flattened, which indicated that the number of reads sequenced in the present study was sufficient to reflect the overall community structure and diversity of rhizosphere fungi. We assigned these reads into OTUs at a 97% similarity threshold, and a total of 3008 OTUs were assigned to all 8 samples (each containing 3 biological duplicates).

### 3.2. α Diversity Indices

**Figure 2:**
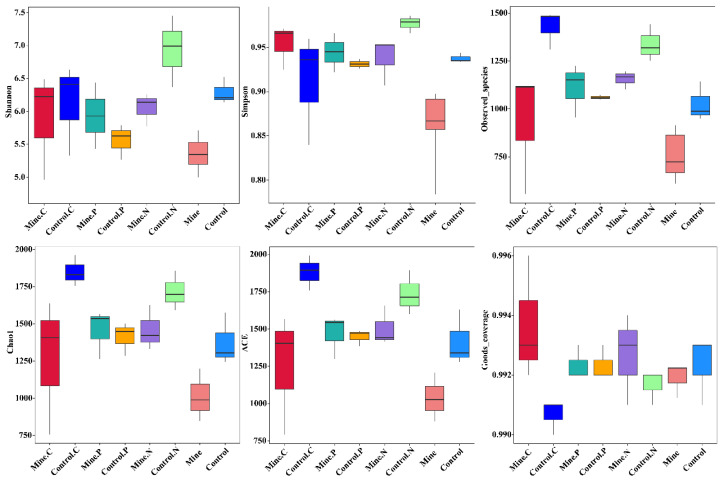
α diversity analysis of different samples.

Compared with bulk soils in nonmining areas, lead zinc mining significantly reduces the fungal diversity index and community richness of bulk soils in mining areas, including the Chao1, ACE, observed species, Simpson, and Shannon indices, which indicates that lead zinc mining significantly affects the fungal diversity and community composition of soils in mining areas (Figure 2). Compared with those in the bulk soil, the fungal diversity and richness of the soil in the three ecologically restored plants were greater. Among them, the Shannon index of *N. reynaudiana*, the observed species index, the Chao 1 index, and the ACE index of *P. cretica* and *N. reynaudiana* significantly improved. In addition, the Simpson index of the rhizosphere soil of the three types of plants in the mining area also significantly improved compared with that of the bulk soil in the mining area. For the same plant species from both mining areas and nonmining areas, the observed species and Simpson and Shannon indices of *N. reynaudiana* from mining areas were significantly lower than those of *N. reynaudiana* from nonmining areas. In addition, the Chao1, ACE, and observed species indices of *C. nubigena* from mining areas also significantly decreased compared with those of the rhizosphere soil of *C. nubigena* from nonmining areas. Most of the community richness and diversity indicators in the rhizosphere soils of the three plant species from the mining and nonmining areas did not significantly differ. For Good’s coverage, the majority of samples had Good’s coverage exceeding 0.99, indicating a good sequencing depth index.

### 3.3. Taxonomic Analyses of Fungal Communities

**Figure 3:**
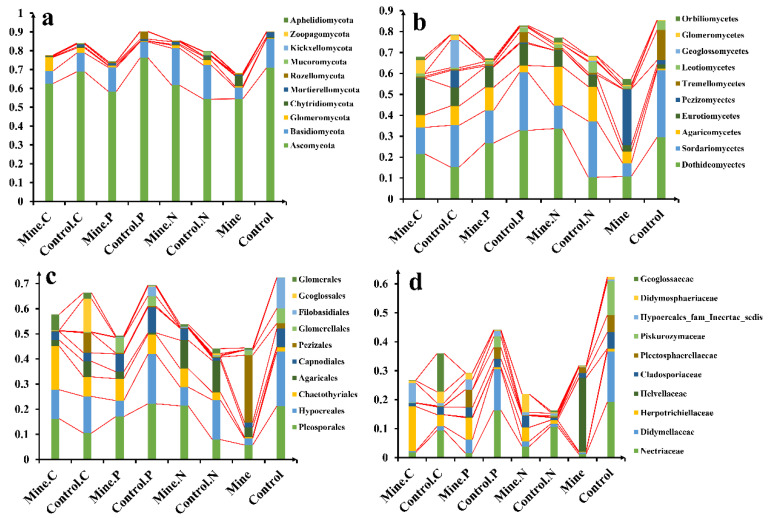
Bar charts of relative abundance of different samples at the phylum (**a**), class (**b**), order (**c**), family (**d**) level.

A total of 16 fungal phyla were identified in all the samples, and the abundances of the 10 most abundant phyla were compared in this study (Figure 3a). We found that Ascomycota was the most abundant phylum in all the samples, accounting for 63.45% of the fungi in all the samples, followed by Basidiomycota (average 12.11%), Glomeromycota (average 2.07%), and Rozellomycota (average 0.88%). Mining activities reduce the abundance of Ascomycota and Basidiomycota in bulk soil but increase the abundance of Glomeromycota and Rozellomycota relative to that in bulk soil in nonmining areas. However, the three ecological restoration plants increased the abundance of Ascomycota and Basidiomycota in the rhizosphere soil relative to that in the bulk soil in the mining area. In addition, the abundance of Ascomycota in the rhizosphere soils of *C. nubigena* and *P. cretica* was significantly lower than that in the control rhizosphere soil from nonmining areas, whereas *N. reynaudiana* increased the abundance of Ascomycota in the mining area soil compared with that in the control rhizosphere soil from nonmining areas.

A total of 69 classes of fungi were detected in all the soil samples (Figure 3b). Dothideomycetes was the most abundant class in all the samples (average 22.58%), followed by Sordariomycetes (average 19.01%), Agaricomycetes (average 8.87%), and Eurotiomycetes (average 8.07%). Mining activities reduce Dothideomycetes and Sordariomycetes and increase the abundance of Agaricomycetes and Eurotiomycetes in bulk soil from mining areas compared with those in bulk soils from nonmining areas. However, the three ecological restoration plants increased the abundance of Dothideomycetes and Sordariomycetes in the rhizosphere soil relative to the bulk soil in the mining area. In addition, the abundance of Dothideomycetes in the rhizosphere soil of *C. nubigena* and *N. reynaudiana* from mining areas was significantly greater than that in the control rhizosphere soil from nonmining areas, whereas *P. cretica* reduced the abundance of Dothideomycetes in the mining area rhizosphere soil compared with that in the control rhizosphere soil from nonmining areas.

Pleosporales (15.27%), Hypocreales (12.47%), Chaetothyriales (6.79%), and Agaricales (5.00%) were the most abundant orders in all the samples (Figure 3c). Compared with nonmining areas, lead zinc mining has reduced the abundance of Pleosporales, Hypocreales, and Chaetothyriales in bulk soil. Compared with that in the bulk soil from the mining area, the abundance of Pleosporales in the rhizosphere soil from the three ecological restoration plants increased. In addition, the abundance of Pleosporales in the rhizosphere soil of *C. nubigena* and *N. reynaudiana* plants from the mining area increased, whereas the abundance of Pleosporales in the rhizosphere soil of *P. cretica* plants decreased relative to their respective control rhizosphere soil samples from nonmining areas.

At the family level, Nectriaceae was the most abundant family in all the samples, followed by Didymellaceae, Herpotrichiellaceae, and Helvellaceae (Figure 3d). We found that the abundances of Nectariaceae and Didymellaceae in the bulk soils from the lead zinc mining areas decreased relative to those in the bulk soils from the nonmining areas. The planting of the three ecological restoration plants significantly increased the abundance of Nectariaceae and Didymellaceae in the soil of the mining area. The abundance of Nectariaceae in the rhizosphere soil of three plants from the mining area significantly decreased compared with that in the control samples from three plants from nonmining areas.

**Figure 4:**
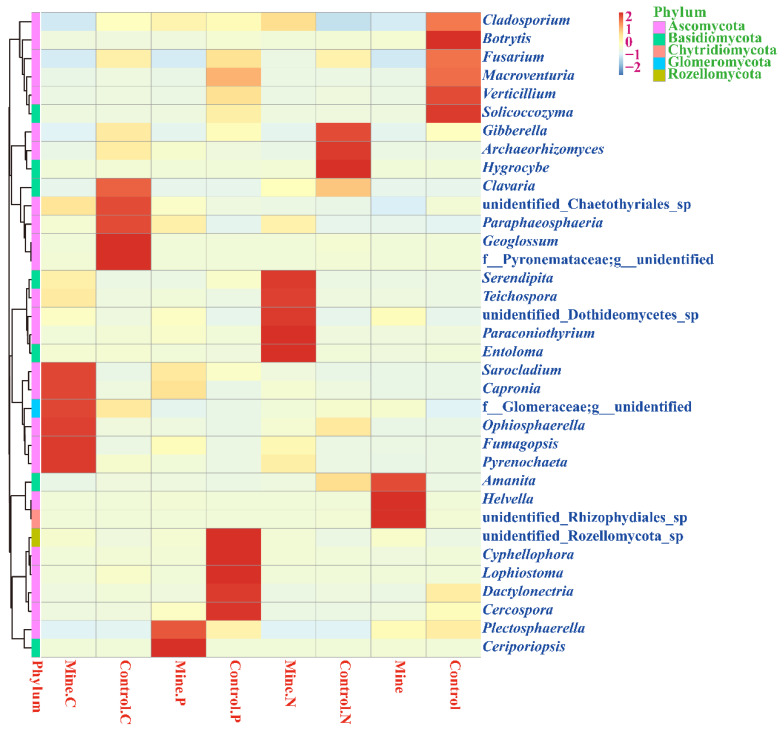
Heatmap analysis of different samples at the genus level, with darker color blocks indicating higher relative abundance of the genus.

At the genus level, *Fusarium* was the most abundant genus among all the samples, followed by *Macroventuria*, *Helvella*, *Cladosporium*, and *Capronia* (Figure 4). We found that the abundances of *Fusarium*, *Macroventuria*, *Cladosporium*, and *Solicocozyma* significantly decreased in the bulk soils from mining areas compared with those in the bulk soils from nonmining areas. In addition, the abundance of *Helvella* significantly increased in bulk soils from mining areas compared with that in bulk soils from nonmining areas. The three plants increased the abundance of *Fusarium* and *Capronia* in the rhizosphere soil relative to the bulk soil in the mining area, whereas the abundance of *Helvella* significantly decreased in the rhizosphere soil of the mining area relative to the bulk soil. The abundance of *Fusarium* and *Macroventuria* in the three plants from the mining area decreased relative to that in the control rhizosphere soil samples from nonmining areas, whereas the abundance of *Capronia* in the three plants from the mining area increased relative to that in the control rhizosphere soil samples from nonmining areas. In addition, different plants enriched different fungal groups in the rhizosphere soil of mining areas relative to those in the rhizosphere soil of nonmining areas. We found that *Capronia* and *Sarocladium* were significantly enriched in the rhizosphere soil of *C. nubigena* plants from mining areas compared with the rhizosphere soil from nonmining areas. *Ceripoliopsis* and *Plectosphaerella* were significantly enriched in the rhizosphere soil of *P. cretica* plants from mining areas compared with the rhizosphere soil from nonmining areas. *Teichospora* and *Serendipita* were significantly enriched in the rhizosphere soil of *N. reynaudiana* plants from mining areas compared with the rhizosphere soil from nonmining areas.

### 3.4. Structural Differentiation of Microbial Communities

**Figure 5:**
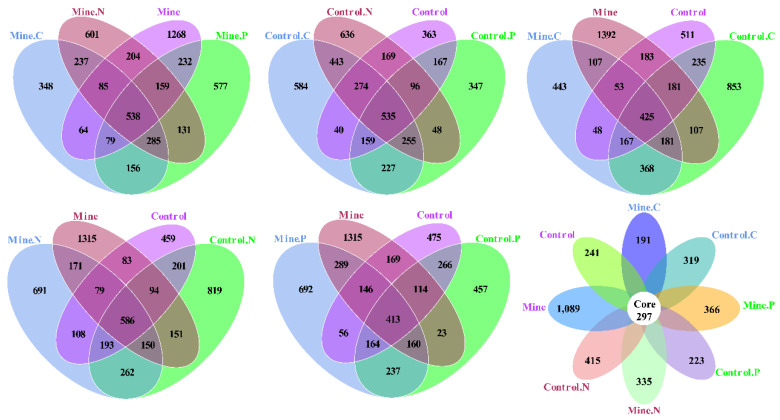
Venn diagram analysis between different samples.

The specific and shared OTUs among different samples were analyzed in this study (Figure 5). For samples from the mining area, Mine. C, Mine. N, Min. P, and Mine contained 348, 601, 577, and 1268 specific OTUs and 538 shared OTUs, respectively. The control samples were collected from nonmining areas. C, Control. N, Control. P, and the control contained 584, 636, 347, and 363 specific OTUs and 535 shared OTUs, respectively. In addition, Mine. C and Control C contained 651 and 1376 specific and 1141 common OTUs, respectively. Mine. N and Control N contained 1049 and 1265 specific and 1191 shared OTUs, respectively. Mine. P and Control P contained 1183 and 860 specific and 974 common OTUs, respectively. The mining of lead and zinc resulted in the production of 1787 specific OTUs and 842 common OTUs in the bulk soil compared with those in the bulk soil from nonmining areas. All the samples generated 191−1089 specific OTUs and 297 shared OTUs.

**Figure 6:**
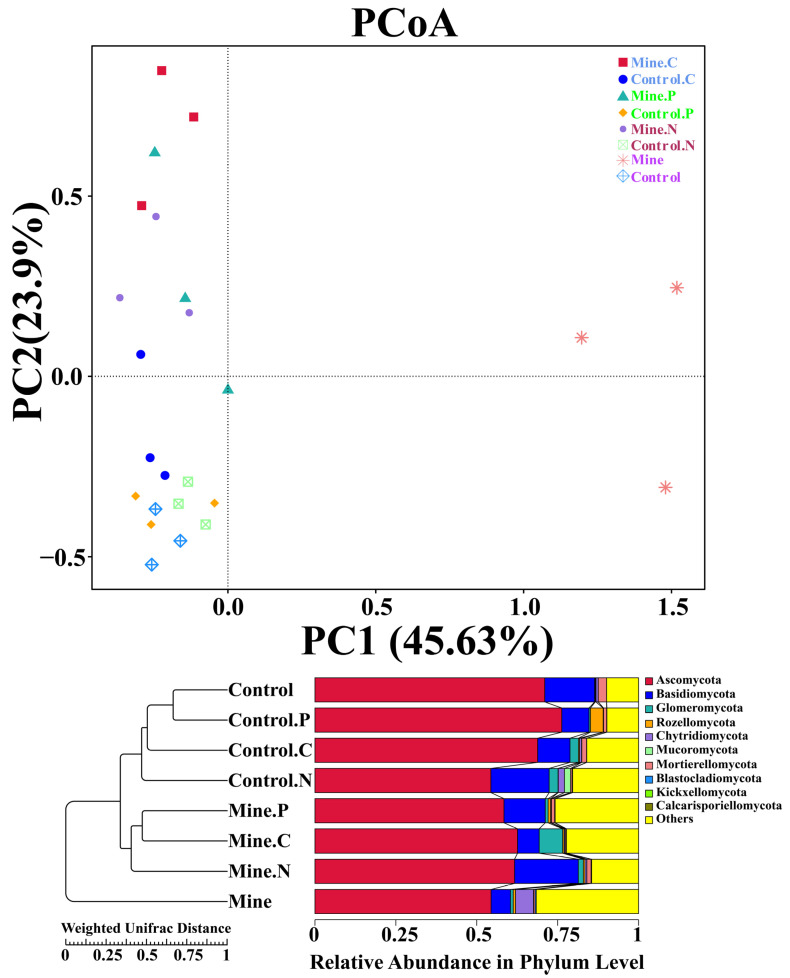
β diversity analysis between different samples based on PCoA and weighted unifrac distance.

The variations in fungal communities among different samples were assessed via PCoA and weighted UniFrac distance analysis (Figure 6). The results revealed that the fungal community structure of the bulk soil samples from the mining area differed significantly from that of the other samples, indicating that lead zinc mining significantly affected the fungal community structure of the soil. After three types of ecologically restored plants were planted, the fungal community structure in the soil was restored to a certain extent, which was similar to the community structure of the control sample. Cluster analysis revealed that the impact of lead zinc mining on the rhizosphere fungal community structure of the three plant species was greater than the impact of the plants themselves on the rhizosphere fungal community structure, indicating the dominant role of lead zinc mining in the ecological restoration of the plant rhizosphere fungal community structure. In addition, the rhizosphere fungal community structure of different plants in mining areas also showed a certain degree of differentiation.

### 3.5. Functional Prediction of the Fungal Community

**Figure 7:**
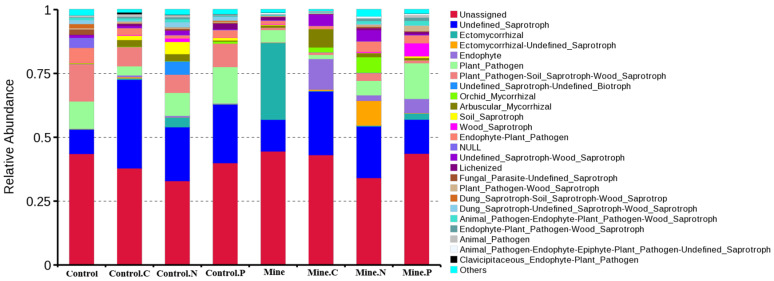
Bar chart of relative abundance of fungal guilds among different samples.

We used FUNGuild to predict the fungal guilds in the soil samples. The fungal functions could be classified into 60 categories, of which unassigned functions accounted for the largest proportion, followed by undefined_Saprotroph, ectomycorrhizal, ectomycorrhizal_undefined_saprotroph, endophyte, and Plant_pathogen. The abundances of different guilds varied among the samples (Figure 7). Compared with bulk soil in nonmining areas, bulk soil in mining areas increased the abundance of undefined_Saprotroph and ectomycorrhizal guilds and decreased the abundance of plant pathogen and plant pathogen-Soil_Saprotroph-Wood_Saprotroph guilds. Compared with the bulk soil in the mining area, the three ecological restoration plants increased the abundance of undefined saprotrophs and plant pathogens in the mining area and decreased the abundance of the Ectomycorrozal guild in the rhizosphere soil from the mining area. In addition, the abundance of undefined saprotrophs in the rhizosphere soil of the three plant species from the mining area decreased, whereas the abundance of endophytes increased relative to that in the rhizosphere soil samples from nonmining areas.

**Figure 8:**
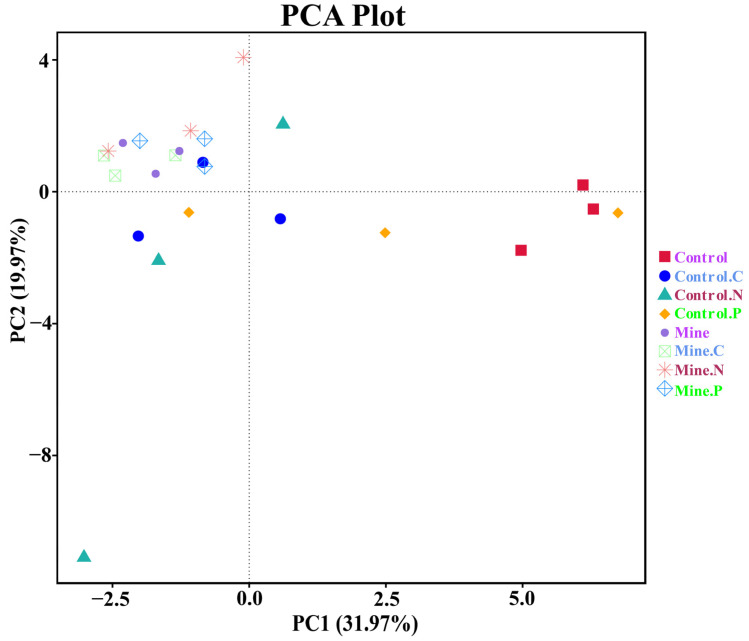
PCA analysis of fungal guilds between different samples.

We further used PCA to assess the variations in fungal guilds among different samples (Figure 8). Compared with the control samples, the lead zinc mining samples led to significant differences in the soil fungal guilds. The planting of ecologically restored plants has to some extent restored the guild of soil fungi, making them more inclined toward the control sample. The guilds of different plants have also undergone a certain degree of differentiation.

## 4. Discussion

This study analyzed the effects of ecological restoration plants on soil fungal communities, diversity, and function in abandoned lead zinc mining areas. The results revealed that lead zinc mines can significantly reduce soil fungal diversity, whereas the planting of three plants restored to the environment improved soil fungal diversity. Fungi in soil play crucial roles in soil material cycling, enhancing plant resistance to biotic and abiotic stresses, promoting plant growth, and nutrient absorption [45,46]. Research has shown that the diversity of soil fungi is closely related to soil quality and plant health [47,48]. This study confirms for the first time that these three plants have significant value in restoring soil quality in lead zinc mining areas. Previous studies have shown that *Carex* plants have strong adaptability to phosphorus deficiency, drought, and waterlogging stress [49]. This study is the first to reveal that *C. nubigena* can be used for ecological restoration in lead zinc mining areas. *P. cretica* has been found to play an important role in the remediation of arsenic and antimony [33,34]. This study is the first to investigate the bioremediation of lead zinc mines, demonstrating the potential of this method for the bioremediation of lead zinc pollution. *N. reynaudiana* has been found to be involved in the bioremediation and detoxification of lead and zinc minerals, demonstrating the widespread application of this plant in the field of lead and zinc remediation [50,51]. In addition, we also found that, compared with those of the same plants from nonmining areas, most community richness and diversity indicators of the three plants from mining areas did not significantly differ, with only a few indicators showing a certain degree of decline, including Chao 1 and ACE. The results showed that after 5 years of cultivation, these three plants were able to adapt well to the environment, confirming their strong adaptability in lead zinc mining areas. In addition, there are certain differences in the diversity and community of rhizosphere fungi among different plants, indicating different adaptation strategies for different plants.

In terms of community composition, lead zinc mining activities significantly reduced the abundance of *Fusarium*, *Macroventuria*, *Cladosporium*, and *Solicocozyma* in the bulk soil, indicating that these fungi are sensitive to lead zinc mining activities. *Fusarium* has been found to have strong tolerance to various heavy metals [52,53], *Cladosporium* is a pathogen of many plants [54,55], and *Solicocozyma* can exist in multiple environments, playing an important role in ecosystems [56,57]. The results revealed that mining activities can cause a certain degree of damage to soil fungal ecosystems. In addition, *Helvella* is significantly more abundant in the bulk soil of mining areas than in that of nonmining areas. The ecological function of *Helvella* is currently unknown. This study is the first to discover that it has strong adaptability to lead zinc mining areas and can be used as a potential resource for the bioremediation of lead zinc mines. In addition, the planting of three ecologically restored plants significantly increased the abundance of *Fusarium* and *Capronia* in the rhizosphere soil of mining areas while reducing the abundance of *Helvella* relative to that in the bulk soil of mining areas, indicating that plants increase their adaptability to the ecological environment through selective fungal community assembly to cope with lead and zinc mining stress [58,59]. In addition, the abundance of *Capronia* also significantly increased in the rhizosphere soil of the three plant species in the mining area compared with the rhizosphere soil of the three plant species planted in nonmining areas. The ecological function of *Capronia* has not yet been elucidated, and research has confirmed for the first time that species of this genus may play important roles in the response of these three plants to lead and zinc mining activities. Different plants also enriched specific fungal populations. For example, *C. nubigena* enriched *Capronia* and *Sarocladium*; *P. cretica* enriched *Ceripoliopsis* and *Plectosphaerella*; and *N. reynaudiana* enriched *Teichospora* and *Serendipita*. This study revealed for the first time that three ecologically restored plants adapted to lead zinc mining activities by specifically and collectively enriching some rhizosphere fungal populations. These common and specific fungal groups help these three plants adapt well to lead zinc mining environments and demonstrate their potential for ecological restoration.

β diversity analysis revealed that mining activities have a significant effect on fungal community structure, leading to significant differences in the soil fungal community. Previous studies have shown that lead zinc mining results in a large amount of heavy metal pollution, which significantly affects the structure of soil bacteria, which is consistent with the results of this study [60,61]. The planting of the three ecological restoration plants led to a certain degree of restoration of the soil fungal community structure, which was similar to the community structure of the control sample. In addition, the impact of lead zinc mining activities on fungal community structure dominated, surpassing the influence of different plants on community structure, indicating the dominant impact of lead zinc mining on the surrounding environment, which is consistent with other research results [62,63]. Fungal guild analysis revealed that lead zinc mining affects the guilds of soil fungi and that planting these three plants has a restorative effect on the guilds of fungi. This study is the first to demonstrate the effects of the planting of ecological restoration plants on the ecological restoration of lead zinc mining areas at the level of fungal guilds in the rhizosphere of plants. In addition, lead zinc mining has led to increases in the Undefined_Saprotroph and Ectomycorrhizal guilds, as well as decreases in the Plant-pathogen and Plant-pathogen-SOil_Saprotroph-Wood_Saprotroph guilds. The planting of three ecologically restored plants has led to an increase in soil undefined_Saprotroph and Plant_pathogen, as well as a decrease in Ectomycorrozal guilds. Endophytes were significantly enriched in the rhizosphere soil of three plants in the mining area compared with the rhizosphere soil of three plants from nonmining areas. Endophytes play important roles in enhancing plant stress resistance, regulating plant growth, and enhancing plant tolerance to heavy metals [64,65]. This study demonstrated that plants adapt to the environment of lead zinc mining areas and restore the ecology of mining areas by selectively selecting and assembling rhizosphere fungal communities and enriching specific guilds. The identified fungal populations and specific rhizosphere fungi-plant combinations provide a reference for the joint ecological restoration of fungal plants.

## 5. Conclusions

In this research, three plant species were chosen for the ecological restoration of abandoned lead zinc mining sites, and the role of soil fungi in assisting plants to adapt to the environment during the restoration process was investigated. This study found that planting ecological restoration plants can significantly increase the fungal diversity of soil in mining areas, and all three ecological restoration plants significantly enriched the abundance of *Capronia*. The guild of endophytes was also significantly enriched in the three types of bioremediation plants. Subsequently, in the process of ecological restoration, we can isolate and screen fungi with high heavy metal tolerance and stable colonization in the rhizosphere of bioremediation plants for microbial plant joint ecological restoration.

## Data Availability

Data are contained within the article and Supplementary Materials.

## Supplementary Materials

The following supporting information can be downloaded at: https://www.mdpi.com/article/10.3390/genes15111398/s1, Table S1: Quality statistics of ITS high-throughput sequencing data.
